# Perception of built environmental factors and physical activity among adolescents in Nigeria

**DOI:** 10.1186/1479-5868-11-56

**Published:** 2014-04-27

**Authors:** Adewale L Oyeyemi, Cornelius M Ishaku, Benedicte Deforche, Adetoyeje Y Oyeyemi, Ilse De Bourdeaudhuij, Delfien Van Dyck

**Affiliations:** 1 Department of Physiotherapy, College of Medical Sciences, University of Maiduguri, Maiduguri, Nigeria; 2 Department of Movement and Sports Sciences, Faculty of Medicine and Health Sciences, Ghent University, Ghent, Belgium; 3 Department of Biometry and Biomechanics, Faculty of Physical Education and Physiotherapy, Vrije Universiteit Brussels, Brussels, Belgium; 4 Research Foundation Flanders, Brussels, Belgium

**Keywords:** Neighborhood environment, Active transport, Youth, Socioeconomic status, Africa

## Abstract

**Background:**

Understanding environmental factors related to adolescents’ physical activity can inform intervention for obesity control and prevention, but virtually no study has been conducted in the African region, where adolescents’ physical inactivity and chronic diseases rates are rising. This study assessed associations between perceived built environmental variables and adolescents’ physical activity (active transportation to school and leisure-time moderate-to- vigorous physical activity), and the moderating effects of neighborhood-level income on association between environmental variables and physical activity among Nigerian boys and girls.

**Methods:**

Participants were 1006 adolescents (12–19 years, 50.4% girls) randomly selected from 11 secondary schools in Maiduguri city, Nigeria. Physical activity and perceptions of environmental characteristics were assessed by validated self-report questionnaires. Separate gender-based, hierarchical multiple moderated linear regression analyses were used to examine the direct associations between the environmental perceptions and physical activity variables (active transportation and leisure-time MVPA; dependent variables), as well as the moderating effects of neighborhood-level income.

**Results:**

Only in boys were direct associations and interaction effect of neighborhood-level income found. Access to destinations was positively associated with active transportation to school (β = 0.18; CI = 0.67, 2.24); while residential density (β = 0.10; CI = 0.01, 1.74) and availability/quality of infrastructures (β = 0.14; CI = 0.49, 2.68) were positively associated with leisure-time MVPA. Also, neighborhood-level income moderated the association between perceived safety and leisure-time MVPA, with more perceived safety related to less MVPA (β = -0.16; CI = -0.01, -0.70) in boys living in high SES neighborhood but marginally related to more MVPA (β = 0.11; CI = -0.04, 2.88, p = 0.06) in boys living in low SES neighborhood.

**Conclusions:**

Few environmental attributes were associated with adolescents’ physical activity in Nigeria. Future studies are needed to determine the multidimensional correlates of physical activity that may be relevant for both adolescents’ boys and girls in Nigeria.

## Background

Regular participation in physical activity in youth has been associated with numerous health benefits [1-3], but adolescence is a period characterized by a steep decline in physical activity levels. The decline in adolescents’ physical activity is more pronounced in older (15–18 years) than younger (12–14 years) adolescents [4-6], in girls than boys [7,8] and in black girls than white girls [5,9]. Also in developing countries, reduced physical activity in adolescents is rapidly becoming common, with physical inactivity prevalence reported to be between 65% and 92% in most African countries [9]. In Nigeria, more than half of school-going adolescents reported low levels of physical activity [10], with attendant rise in the prevalence of overweight (13.8%) and obesity (9.4%) in this age group [11]. It is important to develop effective interventions for the promotion of adolescents’ physical activity in order to prevent and control obesity before it becomes widespread in this age group in Nigeria. However, in order to be effective, interventions should be evidence-based and have potential to reach large number of people [12,13].

World-wide, environmental and policy interventions have been recommended for promoting physical activity because they can influence large groups and bring about population-wide changes [14]. Ecological models of health behaviors, that emphasize the importance of multiple levels of influence to explain health behaviors including physical activity [13], are the most commonly used theoretical framework for understanding such interventions. Within this context, numerous studies have consistently linked the built environments to physical activity, and robust evidence on the relevance of built environmental attributes to physical activity behaviors of adults has emerged [15-19]. However, compared to studies in adults, less research on the relationship of the built environment with physical activity has been conducted among young people, so evidence is still less developed [20-22].

Until now, mixed results have been reported on the associations of numerous environmental attributes with physical activity in adolescents [4,20-24]. A review study that examined four neighborhood environmental factors (access to facilities, availability of facilities, availability of equipment, crime incidence), found availability and access to facilities and equipment to be consistently unrelated to physical activity of adolescents [20]. Objective crime incidence was inversely associated with adolescents’ physical activity in two out of available three studies, but perceived neighborhood safety levels were not associated with adolescents’ physical activity in these studies [20]. In a more recent review, only in 19% of 107 studies that investigated objective built environmental correlates of physical activity of adolescents consistent positive associations were found, with the most consistently supported environmental correlates being mixed land use and residential density [21]. Other objective environmental attributes like access to parks, recreation facilities and street connectivity were inconsistently related with adolescents’ physical activity and no perceived environmental attributes showed a consistent association with both objective and self-reported physical activity [21].

Many of the studies on environmental correlates of adolescents’ physical activity have been conducted in Western high income countries, especially in North America [25-27], Europe [8,24,28,29] and Australia [30-32]. Because developing countries in Africa have specific built environmental features that include diverse terrains, land use, infrastructures, transportation and road designs, and cultural characteristics that may be different from those in Western developed countries, further studies to understand perceived and objective environmental attributes and their associations with adolescents’ physical activity using culturally adapted measures are necessary in Africa. Though cultural adaptation of built environmental surveys to Africa is essential, there is important value in retaining items and constructs that will allow for international comparisons of findings [33]. To date, only in adults studies have been conducted on built environment and physical activity relationships in Africa, and evidence is emerging that favorable perceptions of environmental attributes like increased land used mix, good aesthetics and improved hygienic qualities of neighborhoods and safety from crime and traffic are important for promoting physical activity in Nigerian adults [34-36].

However, evidence from studies conducted in adults may not directly be applicable to adolescents because differences exist in the behaviors and needs of adults and adolescents. Adolescents unlike adults are less independent in their behaviors, and could be more influenced by the environments around home and schools [31,37]. Because the decision of adults on the health seeking behaviors and needs of adolescents may not necessarily reflect the choice of the adolescents, it could be important to independently understand the associations of environmental attributes with physical activity behaviors of adolescents in African countries. Moreover, discerning the perceptions of adolescents in Africa about environmental factors that can influence physical activity could enhance targeted and age specific interventions, aiming to tackle the decline in physical activity during adolescence.

Given that the association between neighborhood environmental variables and physical activity in adolescents has been found to differ by gender [8,38], and to be consistently moderated by neighborhood income [27-29], it is also important to explore subgroup-specific patterns of associations in studies of environmental correlates of physical activity among African adolescents. Until now, no evidence exists among African adolescents, but importantly, patterns of associations of built environmental attributes with physical activity and body weight status were consistently found to be different between African adult men and women [34,35], and between those who reside in low and high socioeconomic status (SES) neighborhoods [39], suggesting that environmental interventions for physical activity promotion in Africa may be gender and neighborhood-income level specific. Since relatively few studies of environmental correlates of physical activity in adolescents have explored the moderating influence of neighborhood-level income or gender on outcomes, insights into how neighborhood income interacts with the associations between built environment and physical activity of male and female adolescents in African countries could contribute to international evidence base and inform local policy on the relevance of gender and area-level income specific interventions for promoting physical activity in the Africa region.

Because built environment characteristics are expected to be strongly related to specific physical activity types (e.g. active transportation or leisure-time physical activity) rather than overall physical activity [12,40], it is important to separately examine the associations between environmental variables and different domains of adolescents’ physical activity. Active transportation and active recreation or sports are two particular and common physical activity behaviors of adolescents that take place in the neighborhood [9,24,28], so these behaviors are particularly important to examine in relation with neighborhood physical environmental attributes [22,41,42]. The aims of the present study were to investigate if perceived neighborhood environmental variables were related to active transportation to school and leisure-time (active sports) physical activity among adolescent boys and girls attending secondary schools in Nigeria, and to investigate whether the association between environmental variables and physical activity is moderated by neighborhood-level income among boys and girls.

## Methods

Eleven secondary schools comprising 5 private and 6 public secondary schools in Maiduguri Metropolitan Council areas were selected to reflect the school types in Nigeria. Maiduguri is the capital and largest city of Borno State in North Eastern Nigeria, with an area of 72.6 km^2^ and a population of about 76,600 inhabitants [43]. Participants were selected using a multistage sampling technique. Initially, each of the available 87 secondary schools in Maiduguri Metropolitan Council were divided into high-income (47 schools) and low-income (40 schools) based on area level socioeconomic status (SES) of their location [43,44], and subsequently stratified by school types (public and private schools). To ensure equal distribution of school types in each SES location, 3 of the 36 available private secondary schools and 2 of the 11 available public secondary schools were randomly selected in the high-income areas. In the low-income areas, 2 private and 4 public secondary schools were randomly selected from the 19 available private and 21 available public secondary schools respectively. All the selected schools agreed to participate in the study. The list of secondary schools in Borno Central Senatorial District accessed from the Ministry of Education in Maiduguri was used to identify and stratify secondary schools into area level income and to construct the sampling frame for random selection of school types.

In the second stage of sampling, one or two classes of about 45 students were randomly selected (lottery method) each from the second to the sixth grade in the 11 selected secondary schools. Thereafter, 20 students were randomly recruited through ballot from each of the selected classes. All students whose names were found in the class attendance register and aged between 12 and 19 years were eligible to be selected and participate in the survey. The first grade of secondary school was not included for participants’ recruitment because students in this class may be younger than 12 years. All participants as well as their parents provided informed consent and approval was obtained from the management of each of the schools before data collection. The study protocol was approved by the Human Research Ethics Committee of the University of Maiduguri Teaching Hospital, Nigeria.

In the selected schools, the potential sample consisted of 3150 students, based on one or two classes per grade (2^nd^ -6^th^) from each of the eleven schools (70 classes in total). In total, 1400 participants (20 students per class) were selected for the study; 1006 participants returned complete and usable surveys, resulting in a response rate of 71.8%. From the selected participants (1400), students who were unwilling or not authorized by their parents to participate (n = 219), were outside the age of 12–19 years (n = 86) or had a disability that prevented independent ambulation (n = 16) were excluded from the study. Seventy three participants did not provide valid data (missing data) on physical activity and built environment variables. No inducement was provided but the students were informed verbally after recruitment and in the cover letter sent to the parents that their responses may assist researchers and Government to identify possible environmental factors that could enhance physical activity behaviors of adolescents in the neighborhoods. Data collection was conducted by a trained team and supervised by the principal investigator. Questionnaires were filled out anonymously by the adolescents themselves, while in the classroom, according to the instructions provided by the supervisor assigned to each of the class room. The survey commenced simultaneously in all the participating classes in a given school. Participants were asked to drop their questionnaires in an envelope placed in front of the class on completion, and subsequently questionnaires were collected by the trained supervisor in the class. Data were collected between February and May, 2011.

## Measures

### Physical activity

The Activity Questionnaire for Adolescents and Young Adults (AQuAA) was used to assess participants’ self-reported active transportation to school and leisure-time moderate-to-vigorous physical activity (MVPA) (sports participation). The AQuAA assesses the frequency (days/week) and duration (min/day) of these physical activity types during the last 7 days. The reliability (ICC = 0.44- 0.59) and validity (rho = 0.05- 0.23) of AQuAA are fair to moderate [45,46], and are comparable to those of other adolescents self-report physical activity questionnaires [47-49]. In a subsample of our participants (n = 56) that were invited to complete a two-week test-retest reliability of the AQuAA, intraclass correlation coefficients of the subscales ranged between ICC = 0.38 and 0.71.

### Environmental perceptions

An adapted version of the Physical Activity Neighborhood Environment Scale (PANES) was used to assess participants’ perceptions of neighborhood environmental factors [50]. A detailed description of the adaptation of the Nigerian version of PANES (PANES-N) has been published [51]. Briefly, the adaptation of PANES for use in Nigeria was undertaken in four phases: revisions by local experts, discussion with international expert, cognitive testing and psychometric evaluations. First, the original PANES was reviewed by eleven experts in Nigeria for adaptation, followed by another revision and subsequent discussion with an international expert who was part of the development of the scale. Next, a cognitive response test was implemented to check the clarity and relevance of PANES’ questions in a small sample of volunteers from different socioeconomic backgrounds. A psychometric study was then conducted in a larger sample to assess the reliability and validity of the modified questionnaire. Neighborhood environmental constructs assessed by the adapted PANES-N included (1) residential density (one question), (2) access to destinations (three questions), (3) connectivity of the street network (one question), (4) infrastructures for physical activity and walking (three questions), (5) social environment (one question), (6) aesthetics (three questions), and (7) neighborhood safety (four questions). With the exception of residential density, all items were phrased as statements about attributes of the neighborhood, with the following response options (four-point scale): strongly disagree, somewhat disagree, somewhat agree, strongly agree.

Response options for the residential density question ranged from single- family detached homes to 13-story or higher apartments (more than 13 apartment/rooms for multiple families). In order to create a summary scale for each of the seven constructs, mean scores were computed for each of the four constructs (scales) with more than one variable (question) and all responses were treated as continuous variables. The PANES-N has good reliability (ICC = 0.62–1.00), and its construct validity was supported by the ability of all it items (only 7 items were significant) to discriminate between environmental features from two neighborhood types selected to be environmentally different [51]. The PANES has also been used previously to evaluate neighborhood environment perceptions in relation to physical activity among adolescents and showed to have good evidence of test-retest reliability in this age group [8,52]. A neighbourhood in the PANES was defined as all the area approximately one kilometer or that could be walked to in 10–15 minutes from the participants’ home.

### Sociodemographic characteristics

A short sociodemographic form was used to obtain information on age, gender, house location, grade of study and parental educational and occupation level (parents’ SES). Information on house location was used to determine the area level SES (neighborhood income) of each participant’s neighborhood, classified as either low-income or high-income neighborhood [43,44]. Information on parents SES (education and occupation) was used to compute participants’ individual level SES [53]. Parental educational status were classified (0–3) as no education, primary education, secondary education and tertiary education, for both father and mother. Responses to questions on parental occupational status were coded (0–3) into unemployed, retired/not working, blue collar job and white collar job, for both father and mother. Parental SES was computed by summing the scores for parental education and occupation to produce a composite scale (0–16) which was further categorized into three groups as low (0–7), middle (8–10) and high (11–16) parental SES [53].

### Data analyses

All analyses were conducted in SPSS 19.0 for Windows. Because the physical activity variables (min/week of active transportation to school and min/week of leisure-time MVPA) were positively skewed, the square root of the original variables was used in the analyses to improve normality. Raw data were used to calculate the descriptive statistics, shown in Table 1. Hierarchical multiple moderated linear regression analyses were conducted to examine the direct associations between the environmental perceptions (seven scales; independent variables) and the different physical activity variables (active transportation and leisure-time MVPA; dependent variables), as well as the moderating effects of neighborhood-level income (high = 1 versus low = 2). All analyses were stratified by gender. Before conducting the analyses, multicollinearity between the independent variables was tested with Pearson correlations. When the correlation coefficient was >0.60, only the predictor with the strongest correlation with the dependent variable was included in the regression model. Individual-level SES, neighborhood-level income and participants’ age and grades of study were always entered as a first block in the regression models in order to control for possible confounding effects. The perceived environmental variables (seven scales) were added as a second block. The cross-product terms of neighborhood-level income × perceived environmental variables (seven cross-products in total) were entered as a third block to examine the potential moderating effects of neighborhood-level income. In case of significant interactions, separate models were run to interpret the direction of the interactions. For all analyses, statistical significance was set at 0.05.

**Table 1:** Descriptive characteristics, physical activity levels and body mass index of participants

	**Total sample (N = 1006)**	**Female (n = 507)**	**Male (n = 499)**	**p-value**^**†**^
**Descriptive characteristics**				
Age (years) Mean (±SD)	15.6 ± 1.7	15.1 ± 1.5	16.1 ± 1.8	<0.001*
Tribe/ethnicity (n, %)				0.200
Igbo	91 (9.0)	54 (10.7)	37 (7.4)	
Hausa/Fulani	131 (13.0)	58 (11.4)	73 (14.6)	
Yoruba	54 (5.4)	30 (5.9)	24 (4.8)	
Kanuri	250 (25.0)	121 (23.9)	130 (26.1)	
Others	479 (47.6)	244 (48.1)	235 (47.1)	
Grade (n, %)				<0.001*
JSS two	255 (25.3)	126 (24.9)	129 (25.9)	
JSS three	302 (30.0)	201 (39.6)	101 (20.2)	
SSS one	133 (13.3)	80 (15.8)	54 (10.8)	
SSS two	191 (19.0)	54 (10.7)	137 (27.5)	
SSS three	124 (12.3)	46 (9.1)	78 (15.6)	
Parents’ SES (n, %)				0.065
Low SES	419 (41.7)	193 (38.1)	226 (45.3)	
Middle SES	311 (31.1)	166 (32.7)	147 (29.5)	
High SES	274 (27.4)	148 (29.2)	126 (25.3)	
Neighborhood SES (n, %)				0.036*
Low SES	703 (69.9)	339 (66.9)	364 (72.9)	
High SES	303 (30.1)	168 (33.1)	135 (27.1)	
**Physical activity and body mass index**				
Active transport				
to school (min/wk),	61.9 ± 110.4	51.4 ± 84.8	72.5 ± 130.6	0.002*
Mean (±SD)				
Leisure MVPA (min/wk),	280.0 ± 350.3	153.9 ± 258.8	408.2 ± 383.3	<0.001*
Mean (±SD)				

^†^Values based on independent t-tests statistics for continuous variables and chi-Square Statistics for categorical variables; ^*^Significant difference between samples (*p* < 0.05).

JSS- Junior Secondary School; SSS- Senior Secondary School; BMI- Body Mass Index;

SES- Socioeconomic status; MVPA- Moderate-to-vigorous physical activity.

## Results

### Descriptive characteristics of the study sample

The final sample comprised of 1006 adolescents (mean age = 15.6 ± 1.72 years; 50.4% female). The majority of the adolescents lived in low-income neighborhoods (69.9%), were in the Junior secondary school grades (2^nd^ and 3^rd^ grades; 55.3%), did not belong to the major ethnic groups (47.6%) and reported lower parental SES (41.7%). On average, the participants reported 61.9 min/wk of active transportation and 280 min/wk of leisure time MVPA, but boys were more physically active than girls in both active transportation (72.5 min/wk vs 51.4 min/wk) and leisure time MVPA (408.2 min/wk vs 153.9 min/wk) (Table 1).

### Environmental correlates of physical activity and moderating effects of neighborhood-level income: results in girls

Before conducting the analyses, multicollinearity between the environmental perceptions was tested. None of the correlation coefficients exceeded r = 0.60 (highest r = 0.24), so all environmental perceptions were included in the regression models.

Neighborhood-level income did not moderate any of the associations of the environmental perceptions with active transportation to school and leisure-time MVPA. Furthermore, none of the environmental perceptions were directly associated with active transportation to school and leisure-time MVPA in girls (Table 2).

**Table 2:** Associations between environmental perceptions and physical activity in girls (n = 507)

	**β**	**95% C.I for β**	**t**	**p**
**Active transportation to school**				
*Interaction effects*				
Neighborhood income ×	-0.13	-2.69, 2.31	-0.59	0.56
Residential density	0.10	-0.99, 1.67	0.50	0.62
Access to destinations	-0.13	-1.13, 0.61	-0.59	0.55
Street connectivity	-0.13	-1.47, 0.88	-0.49	0.62
Social environment	0.41	-0.16, 2.17	1.70	0.09
Infrastructure	-0.20	-1.88, 0.69	-0.91	0.36
Aesthetics	-0.03	-1.51, 1.33	-0.12	0.90
* Safety*				
*Main effects*				
Residential density	0.05	-0.24, 0.72	0.98	0.33
Access to destinations	-0.04	-0.89, 0.37	-0.80	0.43
Street connectivity	0.06	-0.14, 0.69	1.29	0.20
Social environment	-0.01	-0.63, 0.50	-0.22	0.82
Infrastructure	-0.04	-0.80, 0.29	-0.93	0.35
Aesthetics	-0.05	-0.90, 0.29	-1.02	0.31
Safety	-0.05	-0.99, 0.30	-1.09	0.28
**Leisure-time MVPA (sports)**				
*Interaction effects*				
Neighborhood income ×				
Residential density	-0.04	-1.93, 1.60	-0.16	0.86
Access to destinations	0.32	-0.31, 4.27	1.70	0.09
Street connectivity	0.32	-0.40, 2.59	1.44	0.15
Social environment	-0.10	-2.31, 1.73	-0.29	0.78
Infrastructure	0.24	-0.10, 3.02	0.10	0.32
Aesthetics	0.06	-1.89, 2.52	0.28	0.78
Safety	0.18	-1.46, 3.42	0.79	0.43
*Main effects*				
Residential density	0.02	-0.66, 1.00	0.41	0.69
Access to destinations	0.05	-0.54, 1.63	0.98	0.33
Street connectivity	-0.08	-1.28, 0.15	-1.56	0.12
Social environment	0.02	-0.79, 1.17	0.38	0.70
Infrastructure	0.06	-0.37, 1.49	1.18	0.24
Aesthetics	-0.00	-1.05, 0.99	-0.06	0.95
Safety	0.04	-0.71, 1.51	0.71	0.48

MVPA = moderate-to-vigorous physical activity.

*Note:* the regression model was controlled for individual-level SES, neighbourhood level SES, age and grades of study.

### Environmental correlates of physical activity and moderating effects of neighborhood-level income: results in boys

Before conducting the analyses, multicollinearity between the environmental perceptions was tested. None of the correlation coefficients exceeded r = 0.60 (highest r = 0.27), so all environmental perceptions were included in the regression models.

Neighborhood-level income moderated the association between perceived safety and leisure-time MVPA (β = 0.60; CI = 0.63, 6.32). Post hoc analyses showed that in boys living in high-income neighborhoods, more perceived safety was associated with less leisure-time MVPA (β = -0.16; CI = -0.01, -0.70, p = 0.05), while in boys living in low-income neighborhoods, more perceived safety was marginally associated with more leisure-time MVPA (β = 0.11; CI = -0.04, 2.88, p = 0.06) (Not shown in table). Next to this moderating effect, some direct associations were found: access to destinations was positively associated with active transportation to school (β = 0.18; CI = 0.67, 2.24); residential density (β = 0.10; CI = 0.01, 1.74) and availability of infrastructures (β = 0.14; CI = 0.49, 2.68) were positively related to leisure-time MVPA (Table 3).

**Table 3:** Associations between environmental perceptions and physical activity in boys (n = 499)

	**β**	**95% C.I for β**	**t**	**p**
**Active transportation to school**				
*Interaction effects*				
Neighborhood income*				
Residential density	-0.26	-1.95, 0.47	-1.21	0.23
Access to destinations	0.11	-1.50, 2.37	0.44	0.66
Street connectivity	-0.35	-2.09, 0.32	-1.44	0.15
Social environment	-0.12	-1.82, 1.16	-0.44	0.66
Infrastructure	0.30	-0.69, 2.55	1.12	0.26
Aesthetics	-0.21	-2.44, 1.02	-0.80	0.42
Safety	-0.06	-1.99, 1.56	-1.37	0.81
*Main effects*				
Residential density	-0.02	-0.64, 0.45	-0.35	0.73
**Access to destinations**	**0.18**	**0.67, 2.24**	**3.65**	**0.001**
Street connectivity	0.00	-0.52, 0.49	-0.05	0.96
Social environment	-0.02	-0.71, 0.51	-0.32	0.75
Infrastructure	0.01	-0.60, 0.76	0.24	0.81
Aesthetics	0.00	-0.72, 0.77	0.07	0.95
Safety	0.04	-0.49, 1.09	0.74	0.46
**Leisure-time MVPA (sports)**				
*Interaction effects*				
Neighborhood income*				
Residential density	-0.17	-2.73, 1.14	-0.79	0.43
Access to destinations	-0.25	-4.68, 1.54	-1.19	0.24
Street connectivity	-0.27	-3.04, 0.82	-1.04	0.30
Social environment	0.13	-1.79, 2.97	0.50	0.62
Infrastructure	0.29	-1.13, 4.07	1.03	0.31
Aesthetics	-0.02	-3.19, 2.35	-0.03	0.98
**Safety**	**0.60**	**0.63, 6.32**	**2.64**	**0.01**
*Main effects*				
**Residential density**	**0.10**	**0.01, 1.74**	**2.03**	**0.05**
Access to destinations	0.03	-0.91, 1.61	0.55	0.58
Street connectivity	-0.02	-0.99, 0.66	-0.39	0.69
Social environment	0.06	-0.32, 1.64	1.33	0.18
**Infrastructure**	**0.14**	**0.49, 2.68**	**2.84**	**0.01**
Aesthetics	0.06	-0.46, 1.94	1.21	0.23

MVPA = moderate-to-vigorous physical activity.

*Note:* the regression model was controlled for individual-level SES, neighbourhood level SES, age and grades of study.

## Discussion

This study investigated the associations between neighborhood environmental variables and physical activity in a sample of Nigerian adolescents. The main finding was that three of the seven environmental attributes included in the study (access to destinations, residential density and availability of infrastructures) were positively associated with either leisure-time MVPA or active transportation to school only in boys, and none of the environmental attributes were related to either leisure time MVPA or active transportation to school in girls. Neighborhood level income only moderated the association between perceived safety and leisure-time MVPA in boys; no moderating effect of neighborhood income was found for any of the associations between environmental variables and physical activity outcomes in girls.

Based on these findings it can be assumed that the main conclusions from adults’ studies in Nigeria may not be extended to Nigerian adolescents. Favorable perceptions of environmental attributes including high land use mix, good aesthetics, improved neighborhood hygiene and safety form crime and traffic are important environmental attributes that have been consistently linked with higher levels of physical activity in both Nigerian men and women [34-36]. Since mixed and non-comparable results between studies of adolescents and adults have also been found in Western high-income countries [8,20,21,27,28], it could be that worldwide, both in developed and in developing regions, environmental correlates of physical activity in adolescents are potentially different from those of adults.

Because environmental concepts that have been used to define physical activity of adults have commonly been used in studies of adolescents, resulting in inconsistent results, it is possible that these constructs are not very relevant to explain adolescents’ physical activity. The needs to identify specific environmental attributes and to create a unique “activability” index representing those environmental attributes that might enhance the ability of adolescents to be active in the neighborhood were recently emphasized [28]. Based on the present results across countries, it seems that different environmental characteristics are important in adolescents, compared with adults. However, it is possible that for adolescents, the perceptions that parents have of the environment might be more important to explain adolescents’ physical activity levels than the perceptions the adolescents have themselves. It seems that in this age group, a lot of the decisions are still taken by the parents, so this could be important to include in future research. Also, the influence of the physical environment on physical activity levels of adolescents might not be direct and could be mediated by other levels of control including psychosocial factors (e.g., self-efficacy, social support and perceived benefits and barriers) [29,54,55]. Thus, exploring the moderating influence of psychosocial factors on built environment- physical activity associations in adolescents could be an important research direction in the African region.

It is worthy to note that the only significant environmental correlate of active transportation to school in our study was access to destinations and this was only important for boys. Given that the time spent in active transportation by the participants was comparatively lower overall (61.9 min/wk) but significantly higher among boys than girls, it could be that boys rather than girls in Nigeria engage in more active transportation to school when they perceive commonly used destinations (schools, shops to buy things, and bus stops) to be accessible and within easy walking distance from home in their neighborhood. The presence of facilities such as schools, shopping centers, gyms and clubs near adolescents’ home have previously been hypothesized to stimulate walking and cycling as forms of travels among Brazilian male adolescents [38]. Shorter distance (proximity) to school was also an important correlate of active transportation to and from schools among Belgian adolescents [41,55]. However, active transportation among adolescents have also been related with other proximal destinations and facilities (stores, shopping centers, libraries) near adolescents’ homes [38,54], and could be done within the context of leisure and domestic activities rather than to school alone. Thus, it would also be important for future studies in the African region to clarify the potential effect of locating multiple destinations and facilities near adolescents’ home on active transportation of adolescents for different purposes.

The two significant correlates of leisure-time MVPA in male adolescents in this study were residential density and availability of infrastructures. These results confirm the findings of other studies that show significant positive associations between adolescents MVPA and residential density [21] and availability of infrastructures/facilities [30,52,56-58]. However, other studies have also failed to detect significant associations between such variables [8,28,38,54,59]. While the current evidence on associations of environmental variables with adolescents’ leisure-time MVPA remains inconsistent world-wide, the positive association found in the present study is promising, as interventions focusing on the physical environment might be effective for the specific population of Nigerian boys.

Generally, preferences for physical activity behaviors between boys and girls have been reported to be different [60], but the lack of association found between environmental variables and physical activity in girls in our study could be a reflection of the limited opportunities available for girls to use the environment for physical activity in Nigeria. Within the African cultural milieu, girls might be more restricted than boys in their choice of using the environment for physical activity participation because of the pervasive societal gender-construed expectations that often limit their social and behavioral expressions, or parental rules and concerns for safety. Thus, it is possible for girls in our study not to perceive their neighborhood as an important outlet for physical activity behavior. However, given that this is the first study in Nigeria to systematically explore differences between boys and girls in relation to built environmental correlates of physical activity, and that only few studies have generally been conducted in this context [8,38], it may be early at this stage of research to speculate that environmental factors are not relevant for promoting physical activity among Nigerian girls. It is possible that other factors, like psychosocial factors that were not assessed in the present study, are relevant for girls.

An important finding was that neighborhood-level income moderated the association between perceived safety and leisure-time MVPA in boys, with safety negatively associated with leisure-time MVPA among boys living in high-income neighborhood but positively associated among boys in low-income neighborhoods. It is possible that physically active boys in high income neighborhood in Nigeria are more ‘demanding’ about what is safe for them thereby perceiving their neighborhood as less safe for sports related activities, while those not physically active and not coming out of their homes for sporting activities perceive their neighborhood as save since they are never confronted with unsafe situations. Notably, this finding on interaction effects suggests that neighborhood-level income may influence the direction of the association between specific environmental attributes and adolescents’ physical activity. This assumption supports the conclusions of two other studies that have investigated the interactions of neighborhood income on the association between environmental attributes and physical activity in adolescents [27,28]. While in the United States neighborhood walkability was only positively related to physical activity of adolescents in high-income neighborhoods [27], Belgian results showed only a positive association in low-income neighbourhoods [28]. In Nigeria, safety has consistently been associated with physical activity and body weight status of Nigerian adults [34-36], and the present finding further supports the conclusion that safety could be an important environmental correlate, mainly in low-income neighborhoods, exerting stronger influence on health enhancing physical activity in Nigeria than in other countries from the developed nations. Objective crime problems seem to be more dominant in Nigeria with potential consequences for health and well-being of people in this country. Because the socioeconomic reality of a neighborhood may diminish or reinforce the impact of resources offered in the neighborhood [61], it appears that the development of public health interventions that focus on neighborhood crime safety could be an important strategy for promoting physical activity in both Nigerian adults and adolescents (boys).

The results of the present study need to be interpreted in the light of some important limitations. First, the use of cross-sectional data makes it difficult to draw any conclusions on the causality of the associations found. Second, the use of self-reported measures of both physical activity and perception of the environment might have introduced some measurement errors and self-report bias. Third, neighborhoods were defined as the areas around the participants’ homes based on time and distance but recruitment was done in participants’ schools. This definition of neighborhood that combines distance and travel time and un-buffered by boundaries may not be compatible with participants’ own definitions [62], and could have introduced artificial neighborhoods for participants whose schools were located outside the definition imposed for the study. Fourth, studying environmental correlates of physical activity among school going adolescents in Nigeria may produce a sample of adolescents that are high in SES (education) and this may not generalize to the general adolescents’ population in Nigeria. Current estimates indicate that Nigeria has a population of 53.5 million young people aged 10–24 years, the highest in the African region, but only 44% (41% female; 47% male) of this population are enrolled in secondary school [63]. Fifth, the clustered nature of the data presented in this study was not accounted for during data analysis. Ignoring the presence of clustering is a serious issue that compromises the analytical quality and robustness of outcomes in built environment studies [64]. We regret that this clustering variable was not available in our datafiles. It is important that future studies in the African region should evaluate the relationships between environmental variables and physical activity using an analytical approach that takes clustering effects into consideration. The strengths of this study include the examination of gender-specific associations using valid and established scales. The large representative sample of secondary school students could enhance the generalization of results to this population. The focus on an African population is important and unique given the rising prevalence of adolescents’ obesity in this region and the predominance of data from Western high income countries. Identifying important environmental constructs for adolescents’ physical activity in the African region would not only help explain differences between adults and adolescents studies but could also enhance robust and age-specific evidence acquisition necessary to develop effective interventions.

In conclusion, we found that a limited number of environmental attributes were associated with adolescents’ physical activity in Nigeria, with only three out of seven environmental characteristics being associated with physical activity in boys only. Only one interaction effect of neighborhood income was found, with perceptions of safety being positively associated with physical activity among adolescents in low-income neighborhoods, suggesting that the physical environment across high and low income neighborhoods may not be strongly related to physical activity in Nigerian adolescents. However, the present study underscores the need for future research assessing the multidimensional correlates of adolescents’ physical activity, like psychosocial factors (e.g., self-efficacy, social support and perceived benefits and barriers), that may be important to explain physical activity of adolescents before conclusions can be made on the relevance of designing built environmental intervention for this age group in Nigeria.

## Competing interests

Authors declare there is no competing financial interest associated with this study.

## Authors’ contributions

ALO conceived and designed the study; drafted the manuscript; was involved with statistical analysis and interpretation of data; and contributed to data acquisition. CMI and AYO contributed to study design, data acquisition and preparation, and participated in critical revision of the drafted manuscript. BD and IDB participated in the design of the study, interpretation of data and critically revised the drafted manuscript for important intellectual content. DVD contributed to study design, conducted the statistical analysis and interpretation of data, helped to draft the data analysis and result sections, and participated in critical revision of the drafted manuscript. All authors read and approved the final manuscript.

## References

[B1] Schmalz DL Deane GD Birch LL Davison KK A longitudinal assessment of the links between physical activity and self-esteem in early adolescent non-Hispanic females Publ Soc Adolesc Med 2007 41 559 565 10.1016/j.jadohealth.2007.07.001 PMC2562306 18023784

[B2] World Health Organization Global recommendations on physical activity for health 2010 Geneva: World Health Organization 26180873

[B3] Fedewa AL Ahn S The effects of physical activity and physical fitness on children's achievement and cognitive outcomes: a metaanalysis Res Q Exerc Sport 2011 82 521 535 10.1080/02701367.2011.10599785 21957711

[B4] Sallis JF Prochaska JJ Taylor WC A review of correlates of physical activity of children and adolescents Med Sci Sports Exerc 2000 32 963 975 10795788 10.1097/00005768-200005000-00014

[B5] Kimm SYS Glynn NW Kriska AM Barton BA Kronsberg SS Daniels SR Crawford PB Sabry ZI Liu K Decline in physical activity in black girls and white girls during adolescence N Eng J Med 2002 347 10 709 715 10.1056/NEJMoa003277 12213941

[B6] Troiano RP Berrigan D Dodd KW Masse LC Tilert T McDowell M Physical activity in the United States measured by accelerometer Med Sci Sports Exerc 2008 40 1 181 188 10.1249/mss.0b013e31815a51b3 18091006

[B7] Butcher K Sallis JF Mayer JA Woodruff S Correlates of physical activity guideline compliance for Adolescents in 100 U.S. Cities J Adolesc Health 2008 42 4 360 368 10.1016/j.jadohealth.2007.09.025 18346661 PMC2293305

[B8] Santos MP Page AS Cooper AR Ribeiro JC Mota J Perceptions of the built environment in relation to physical activity in Portuguese adolescents Health Place 2009 15 548 552 10.1016/j.healthplace.2008.08.006 19004663

[B9] Guthold R Cowan MJ Autenrieth CS Kann L Riley LM Physical activity and sedentary behavior among schoolchildren: a 34-country comparison J Pediatr 2010 157 1 42 49 10.1016/j.jpeds.2010.01.023 20304415

[B10] Adeniyi AF Okafor NC Adeniyi CY Depression and physical activity in a sample of Nigerian adolescents: levels, relationships and predictors Child Adolescent Psychiatr Ment Health 2011 5 16 10.1186/1753-2000-5-16 PMC3117803 21569581

[B11] Oduwole AA Ladapo TA Fajolu IB Ekure EN Adeniyi OF Obesity and elevated blood pressure among adolescents in LagosNigeria: a cross-sectional study BMC Public Health 2012 12 616 10.1186/1471-2458-12-616 22867531 PMC3490830

[B12] Sallis JF Cervero R Ascher WW Henderson K Kraft MK Kerr J An ecological approach to creating active living communities Annu Rev Public Health 2006 27 14.1 14.26 10.1146/annurev.publhealth.27.021405.102100 16533119

[B13] Sallis JF Owen N Fisher EB Glanz K, Rimer BK, Viswanath K Ecological models of health behavior Health behavior and health Education: Theory, research, and practice 2008 4 San Francisco: Jossey-Bass 465 486

[B14] World Health Assembly 57.17 Global strategy on diet and physical activity 2004 Geneva: World Health Organization 10.1177/156482650402500310 15460274

[B15] Bauman AE Reis RS Sallis JF Wells JC Loos RJF Martin BW Correlates of physical activity: Why are some people physically active and others not? Lancet 2012 380 31 44 10.1016/S0140-6736(12)60735-1 22818938

[B16] Owen N Humpel N Leslie E Bauman A Sallis JF Understanding environmental influences on walking: review and research agenda Am J Prev Med 2004 27 67 76 10.1016/j.amepre.2004.03.006 15212778

[B17] Saelens BE Handy SL Built environment correlates of walking: a review Med Sci Sports Exerc 2008 40 s550 s566 10.1249/MSS.0b013e31817c67a4 18562973 PMC2921187

[B18] Gebel K Bauman AE Petticrew M The physical environment and physical activity: a critical appraisal of review articles Am J Prev Med 2007 32 361 369 10.1016/j.amepre.2007.01.020 17478260

[B19] Van Holle V Deforche B Van Cauwenberg J Goubert L Maes L Van de Weghe N De Bourdeaudhuij I Relationship between the physical environment and different domains of physical activity in European adults: a systematic review BMC Public Health 2012 12 807 10.1186/1471-2458-12-807 22992438 PMC3507898

[B20] Ferreira I van der Horst K Wendel-Vos W Kremer S Van Lenthe FJ Brug J Environmental correlates of physical activity in youth—a review and update Obes Rev 2007 8 129 154 10.1111/j.1467-789X.2006.00264.x 17300279

[B21] Ding D Sallis JF Kerr J Lee S Rosenberg DE Neighborhood environment and physical activity among youth: a review Am J Prev Med 2011 41 4 442 455 10.1016/j.amepre.2011.06.036 21961474

[B22] Panter JR Jones AP van Sluijs EMF Environmental determinants of active travel in youth: A review and framework for future research Int J Behav Nutr Phys Act 2008 5 34 10.1186/1479-5868-5-34 18573196 PMC2483993

[B23] Davison KK Lawson CT Do attributes in the physical environment influence children’s physical activity? A review of the literature Int J Behav Nutr Phys Act 2006 3 19 10.1186/1479-5868-3-19 16872543 PMC1557665

[B24] Van Dyck D Cardon G Deforche B De Bourdeaudhuij I Lower neighbourhood walkability and longer distance to school are related to physical activity in Belgian adolescents Prev Med 2009 48 516 518 10.1016/j.ypmed.2009.03.005 19285102

[B25] Kligerman M Sallis JF Ryan S Frank LD Nader PR Association of neighborhood design and recreation environment variables with physical activity and body mass index in adolescents Am J Health Promot 2007 21 4 274 277 10.4278/0890-1171-21.4.274 17375494

[B26] Evenson KR Birnbaum AS Bedimo-Rung AL Sallis JF Voorhees CC Ring K Elder JP Girls’ perception of physical environmental factors and transportation: reliability and association with physical activity and active transport to school Int J Behav Nutr Phys Act 2006 3 28 10.1186/1479-5868-3-28 16972999 PMC1594571

[B27] Kerr J Rosenberg D Sallis JF Saelens BE Frank LD Conway TL Active commuting to school: Associations with environment and parental concerns Med Sci Sports Exerc 2006 38 787 794 10.1249/01.mss.0000210208.63565.73 16679998

[B28] De Meester F Van Dyck D De Bourdeaudhuij I Deforche B Sallis JF Cardon G Active living neighborhoods: is neighborhood walkability a key element for Belgian adolescents? BMC Public Health 2012 12 7 10.1186/1471-2458-12-7 22216923 PMC3265406

[B29] De Meester F Van Dyck D De Bourdeaudhuij I Deforche B Cardon G Do psychosocial factors moderate the association between neighborhood walkability and adolescents’ physical activity? Soc Sci Med 2013 81 1 9 23422054 10.1016/j.socscimed.2013.01.013

[B30] Hume C Salmon J Ball K Children’s perceptions of their home and neighborhood environments, and their association with objectively measured physical activity: a qualitative and quantitative study Health Educ Res 2005 20 1 13 15253992 10.1093/her/cyg095

[B31] Carver A Timperio AF Crawford D Neighborhood road environments and physical activity among youth: the CLAN study J Urban Health 2008 85 4 532 544 10.1007/s11524-008-9284-9 18437579 PMC2443253

[B32] Timperio A Ball K Salmon J Roberts R Giles-Corti B Simmons D Baur LA Crawford D Personal, family, social, and environmental correlates of active commuting to school Am J Prev Med 2006 30 45 51 10.1016/j.amepre.2005.08.047 16414423

[B33] Kerr J Sallis JF Owen N De Bourdeaudhuij I Cerin E Reis R Sarmiento O Frömel K Mitáš J Troelsen J Christiansen LB Macfarlane D Salvo D Schofield G Badland H Guillen-Grima F Aguinaga-Ontoso I Davey R Bauman A Saelens BE Riddoch C Ainsworth B Pratt M Schmid T Frank LD Adams MA Conway TL Cain K Van Dyck D Bracy B Advancing Science and Policy through a Coordinated International Study of Physical Activity and Built Environments: IPEN Methods J Phys Act Health 2013 10 581 601 22975776 10.1123/jpah.10.4.581

[B34] Oyeyemi AL Adegoke BOA Oyeyemi AY Sallis JF Perceived environmental correlates of physical activity and walking in African young adults Am J Health Promot 2011 25 5 e10 e19 10.4278/ajhp.090918-QUAN-304 21534826

[B35] Oyeyemi AL Sallis JF Adegoke BOA Oyeyemi AY De Bourdeaudhuij I Perception of neighborhood safety is related with physical activity among adults in Nigeria BMC Public Health 2012 12 294 10.1186/1471-2458-12-294 22520066 PMC3355030

[B36] Oyeyemi AL Sallis JF Deforche B Oyeyemi AY De Bourdeaudhuij I Van Dyck D Evaluation of the Neighborhood Environment Walkability Scale in Nigeria Int J Health Geogr 2013 12 16 10.1186/1476-072X-12-16 23514561 PMC3606828

[B37] Haug E Torsheim T Sallis JF Samdal O The characteristics of the outdoor school environment associated with physical activity Health Educ Res 2010 25 2 248 256 10.1093/her/cyn050 18936270 PMC2839138

[B38] de Farias JJC de Silva LA Mota J Santos MP Ribeiro JC Hallal PC Perception of the social and built environment and physical activity among Northeastern Brazil adolescents Prev Med 2011 52 114 119 10.1016/j.ypmed.2010.12.002 21147155

[B39] Oyeyemi AL Adegoke BOA Oyeyemi AY Deforche B De Bourdeaudhuij I Sallis JF Environmental factors associated with overweight among adults in Nigeria Int J Behav Nutr Phys Act 2012 9 32 10.1186/1479-5868-9-32 22452904 PMC3331819

[B40] Giles-Corti B Timperio A Bull F Pikora T Understanding physical activity environmental correlates: increased specificity for ecological models Exerc Sport Sci Rev 2005 33 175 181 10.1097/00003677-200510000-00005 16239834

[B41] De Meester F Van Dyck D De Bourdeaudhuij I Deforche B Cardon G Does the perception of neighborhood built environmental attributes influence active transport in adolescents? Int J Behav Nutr Phys Act 2013 10 38 10.1186/1479-5868-10-38 23531272 PMC3618145

[B42] Rosenberg D Ding D Sallis JF Kerr J Norman GJ Durant N Harris SK Saelens BE Neighborhood environment walkability scale for youth (NEW-Y): reliability and relationship with physical activity Prev Med 2009 4 213 218 19632263 10.1016/j.ypmed.2009.07.011

[B43] National Bureau of Statistics Statistical Fact Sheet, 2008 Federal Republic of Nigeria Available at: http://www.nigerianstat.gov.ng/ (accessed January 25, 2011)

[B44] National Population Commission (NPC) [Nigeria] and ICF Macro Nigeria Demographic and Health Survey 2003 2004 Abuja, Nigeria: National Population Commission and ICF Macro

[B45] Chinapaw MJM Slootmaker SM Schuit AJ van Zuidam M van Mechelen W Reliability and validity of the Activity Questionnaire for Adults and Adolescents (AQuAA) BMC Med Res Methodol 2009 9 58 10.1186/1471-2288-9-58 19664254 PMC3224705

[B46] Liu RD Buffart LM Kersten MJ Spiering M Brug J van Mechelen W Chinapaw MJ Psychometric properties of two physical activity questionnaires, the AQuAA and the PASE, in cancer patients BMC Med Res Methodol 2011 11 30 10.1186/1471-2288-11-30 21410979 PMC3079697

[B47] Hong TK Trang NH van der Ploeg HP Hardy LL Dibley MJ Validity and reliability of a physical activity questionnaire for Vietnamese adolescents Int J Behav Nutr Phys Act 2012 9 93 10.1186/1479-5868-9-93 22853177 PMC3487813

[B48] Hagstromer M Bergman P De Bourdeaudhuij I Ortega FB Ruiz JR Manios Y Rey-Lopez JP Phillip K von Berlepsch J Sjostrom M HELENA Study Group Concurrent validity of a modified version of the International Physical Activity Questionnaire (IPAQ-A) in European adolescents: The HELENA Study Int J Obes 2008 32 S5 S42 S48 10.1038/ijo.2008.182 19011653

[B49] Ottevaere C Huybrechts I De Bourdeaudhuij I Sjöström M Ruiz JR Ortega FB Hagströmer M Widhalm K Molnár D Moreno LA Beghin L Kafatos A Polito A Manios Y Mártinez-Gómez D De Henauw S Comparison of the IPAQ-A and actigraph in relation to VO2max among European adolescents: the HELENA study J Sci Med Sport 2011 14 317 324 10.1016/j.jsams.2011.02.008 21444243

[B50] International Physical Activity Prevalence Study Self- administered environmental module. Revised November 2002 Available at: http://sallis.ucsd.edu/Documents/IPAQIPS.pdf. Accessed March 3, 2012

[B51] Oyeyemi AL Sallis JF Oyeyemi AY Mahmud-Amin M De Bourdeaudhuij I Deforche B Adaptation, test-retest reliability, and construct validity of the physical activity neighborhood environment scale in Nigeria J Phys Act Health In press 10.1123/jpah.10.8.1079 23220861

[B52] Mota J Almeida M Santos P Ribeiro JC Perceived neighborhood environments and physical activity in adolescents Prev Med 2005 41 834 836 10.1016/j.ypmed.2005.07.012 16137754

[B53] Doku D Koivusilta L Rimpela A Indicators for measuring material affluence of adolescents in health inequality research in developing countries Child Indic Res 2010 3 243 260 10.1007/s12187-009-9045-7 20339572 PMC2837228

[B54] Deforche B Van Dyck D Verloigne M De Bourdeauduij I Perceived social and physical environmental correlates of physical activity in older adolescents and the moderating role of self efficacy Prev Med 2010 50 Suppl. 1 S24 S29 19818363 10.1016/j.ypmed.2009.08.017

[B55] Van Dyck D De Bourdeaudhuij I Cardon G Deforche B Criterion distances and correlates of active transportation to school in Belgian older adolescents Int J Behav Nutr Phys Act 2010 7 87 10.1186/1479-5868-7-87 21143868 PMC3004815

[B56] Normal GJ Nutter SK Ryan S Sallis JF Calfas KJ Patrick K Community design and access to recreational facilities as correlates of adolescents physical activity and body-mass index J Phys Act Health 2006 3 S118 S128 10.1123/jpah.3.s1.s118 28834510

[B57] Huang SJ Hung WC Sharpe PA Wai JP Neighbourhood environment and physical activity among urban and rural school children in Taiwan Health Place 2010 16 470 476 10.1016/j.healthplace.2009.12.004 20137996

[B58] Tucker P Irwin JD Gilliland J He M Larsen K Hess P Environmental influences on physical activity levels in youth Health Place 2009 15 357 363 10.1016/j.healthplace.2008.07.001 18706850

[B59] Hager ER Witherspoon DO Gormley C Latta LW Pepper MR Black MM The perceived and built environment surrounding urban schools and physical activity among adolescent girls Ann Behav Meddoi:10.1007/s12160-012-9430-1. Published online: 19 January 2013 10.1007/s12160-012-9430-1 PMC3609655 23334761

[B60] Kuo J Schmitz KH Evenson KR McKenzie TL Jobe JB Rung AL Gittelsohn J Pate RR Physical and social contexts of physical activities among adolescent girls J Adolesc Health 2009 6 144 152 10.1123/jpah.6.2.144 PMC4959469 19420391

[B61] Bernard P Charafeddine R Frohlich KL Daniel M Kesten Y Potvin L Health inequalities and place: a theoretical conception of neighborhood Soc Sci Med 2007 65 9 1839 1852 10.1016/j.socscimed.2007.05.037 17614174

[B62] Smith G Gidlow C Davey R Foster C What is my walking neighborhood? A pilot study of English adults’ definitions of their local walking neighborhoods Int J Behav Nutr Phys Act 2010 7 34 10.1186/1479-5868-7-34 20459636 PMC2873577

[B63] Population Reference Bureau The World’s Youth 2013 data sheet http://www.prb.org/Publications/Datasheets/2013/youth-datasheet-2013.aspx/. Accessed January 27, 2014

[B64] Cerin E Statistical approaches to testing the relationships of the built environment with residents-level physical activity behaviour and health outcomes in cross-sectional studies with cluster sampling J Plann Lit 2011 26 151 167 10.1177/0885412210386229

